# Active PKG II inhibited the growth and migration of ovarian cancer cells through blocking Raf/MEK and PI3K/Akt signaling pathways

**DOI:** 10.1042/BSR20190405

**Published:** 2019-08-13

**Authors:** Yan Wu, Qin Cai, Wei Li, Zhensheng Cai, Ying Liu, Hongfan Li, Ji Pang, Yongchang Chen

**Affiliations:** 1 Department of Physiology, School of Medicine, Jiangsu University, Zhenjiang 212013, China; 2 The Fourth Affiliated Hospital of Jiangsu University, Zhenjiang 212001, China

**Keywords:** PKG II, L-arginine, proliferation, migration, apoptosis, ovarian cancer

## Abstract

Despite advances in chemotherapy, ovarian cancer (OC) is still the most lethal gynecologic malignancy. So, it is imperative to explore its mechanism and find novel targets to improve the outcome. Type II cyclic guanosine 3′,5′-monophosphate (cGMP)-dependent protein kinase (PKG II) has been recently reported to inhibit proliferation and metastasis in several tumors. The present study is to clarify the effect of PKG II combined with l-arginine (l-Arg) on OC cells. SKOV3 and A2780 cells were infected with adenovirus coding cDNA of PKG II to increase PKG II expression and l-Arg was applied to activate this kinase. CCK8 assay, Transwell migration and TUNEL assay were applied to detect the proliferation, migration and apoptosis of the OC cells, respectively. Western blotting was used to detect the level of total and phosphorylated proteins. Our results showed that co-treatment with PKG II and l-Arg inhibited EGF-induced proliferation and the expression of Proliferating Cell Nuclear Antigen (PCNA), Cyclin E and N-Cadherin, whereas up-regulated the expression of E-Cadherin, abolished the anti-apoptotic effect of EGF, prevented the process of epithelial-to-mesenchymal transition (EMT) as well as blocked EGF-triggered Raf-MEK and phosphatidylinositol 3-kinase (PI3K)/Akt signaling pathways. Our results suggested that PKG II activated by l-Arg could inhibit proliferation and migration and promote the apoptosis of OC cells. Based on the above results and our previous data, it is speculated that PKG II is an inhibitor of cancer with extensive effects.

## Introduction

Among the different gynecological cancers, ovarian cancer (OC) is the most lethal gynecological malignancy in developed countries [1,2], which is associated with late diagnosis due to the lack of specific symptoms, as well as the high relapse rate after treatment with surgery or chemotherapy. Although chemotherapy is initially effective for most patients, approximately 70% of women with advanced OC are still dead owing to the drug resistance [3,4]. Hence, it is needed to find novel therapeutic methods to improve clinical outcomes of ovarian malignancy, especially, biological therapy targeting novel checkpoint molecule.

Cyclic guanosine 3′,5′-monophosphate (cGMP)-dependent protein kinases (PKG) are serine/threonine kinases in mammalian cells and consist of two types, cytosolic PKG I and membrane-bound PKG II [5]. PKG I and PKG II have distinct subcellular localization, tissue expression, and substrates [6–9]. Membrane-bound PKG II is a key regulator of bone growth, renin secretion, and memory formation [10]. It phosphorylates several downstream substrates and acts as a major regulator for them, including cystic fibrosis transmembrane conductance regulator (CFTR) and α-amino-3-hydroxy-5-methyl-4-isoxazolepropionic acid (AMPA) receptor [8,11,12]. Furthermore, accumulated data suggested that PKG II is involved in inhibiting proliferation and inducing apoptosis in several kinds of cells, such as prostatic stromal cells, vascular smooth muscle cells and endothelial cells [13–15]. Recently, PKG II was reported to negatively regulate fibroblast growth factor (FGF)-mediated signaling by phosphorylation Raf-1 at Ser^43^ in rat chondrosarcoma cells [16]. Our previous study also indicated that PKG II inhibited the development of gastric cancer cells via blocking EGF/HGF-induced proliferation and migration of gastric and hepatic cancer cells [17–20], as well as blocked LPA-induced cell migration [21,22]. However, it remains unclear whether there is an more extensive antitumor effect for PKG II in other tumor cells, such as gynecological tumor. Moreover, the antitumor effects of PKG II were dependent on the activation induced by cGMP or cGMP analog 8-pCPT-cGMP [17]. So, it also needs to be explored whether PKG II could be effectively activated by other substrates. Published data indicated that nitric oxide (NO) allosterically interacts with soluble guanylyl cyclase (sGC) to catalyze the synthesis of the second messenger cGMP, which in turn activates cGMP-dependent PKG [23] and/or other effector proteins, including ion channels, pumps, and phosphodiesterases (PDEs) [24]. As an important bio-regulatory molecule, NO could be synthesized from l-arginine (l-Arg) through a catalytic reaction catalyzed by NO synthase (NOS) [25]. Therefore, as an NO precursor, l-Arg might have the potential to activate PKG II via synthesizing NO and then inducing sGC to generate cGMP. In fact, considering the complex functions of l-Arg, it is still needed to confirm whether l-Arg can activate PKG II and help the kinase to perform its biological functions. To illustrate the above speculation, we studied the effect of l-Arg on the activation of PKG II using OC cell model. Our results suggest that l-Arg-triggered PKG II activation inhibited EGF-induced Raf-MEK and phosphatidylinositol 3-kinase (PI3K)/Akt signaling pathways, indicating that l-Arg could benefit the antitumor effect of PKG II in OC cells by activating this kinase instead of cGMP.

## Materials and methods

### Cell lines and reagents

Human OC cell lines A2780 and SKOV3 were purchased from CHI Scientific Inc. (Maynard, MA, U.S.A.) and Institute of Cell Biology (Shanghai, China), respectively. Adenoviral vectors encoding the cDNA of β-galactosidase (Ad-LacZ) and PKG II (Ad-PKG II) were kind gifts from Dr. Gerry Boss and Dr. Renate Pilz, University of California, San Diego, CA, U.S.A. Dulbecco’s modified Eagle’s medium (DMEM) and FBS were obtained from Gibco (Grand Island, NY, U.S.A.). The primary antibodies against Bcl-2, Bax, Proliferating Cell Nuclear Antigen (PCNA), Cyclin E, E-Cadherin, N-Cadherin, phospho (p-) and total EGFR, ERK, c-Raf, MEK, PI3K, and Akt were obtained from Cell Signaling Technology (Danvers, MA, U.S.A.). Antibodies against PKG II, cleaved-Caspase 3 and β-actin were from Santa Cruz (Dallas, TX, U.S.A.). The antibody against β-galactosidase was from Proteintech (Proteintech Group, Inc, Rosemont, IL, U.S.A.). Electrochemiluminescence (ECL) reagents were purchased from Millipore (Billerica, MA, U.S.A.).

### Cell culture and infection with adenoviral vectors

The cells were cultured in DMEM containing 10% FBS at 37°C in 5% CO_2_. The medium was changed every 2 days and the cells were subcultured at confluence. On the day before infection with adenovirus, cells were freshly planted into six-well plates at 70–80% confluence, and infected with Ad-LacZ or Ad-PKG II for 24 h, serum-starved for 12 h, followed by treatment with l-Arg and EGF.

### Western blotting

The treated cells were rapidly lysed in Cell lysis buffer for Western blot and IP (P0013, Beyotime, Beijing, China) on ice and then centrifuged at 12000×***g***, 30 min, 4°C. The supernatant was collected and the sample proteins were separated by 8–12% sodium dodecyl sulfate/polyacrylamide gel electrophoresis (SDS/PAGE), and transferred to a polyvinylidene fluoride (PVDF) membrane. The PVDF membranes were blocked with 5% (w/v) non-fat milk in Tris-buffered saline, 0.1% Tween-20 (TBS-T) for 1 h at room temperature and incubated with the primary antibody at 4°C overnight, followed by incubation with the corresponding secondary antibody at room temperature for 1 h. ECL reagents were applied to show the positive bands on the membrane.

### TUNEL assay

A total of 5 × 10^4^ cells were seeded in 24-well plates, infected with Ad-PKG II for 24 h, and treated with l-Arg (2mM, 4 h) and EGF (100ng/ml, 24 h). The cells were then fixed and detected using the *In Situ* Cell Death Detection kit from Roche Diagnostics, according to the manufacturer’s protocol (Mannheim, Germany, Cat. 11684817910). Briefly, the cells on the glass slides were fixed with 4% paraformaldehyde solution, rinsed with PBS, incubated with 3% H_2_O_2_ in methanol, rinsed with PBS, incubated in permeabilisation solution for 2 min on ice, and then incubated with TUNEL reaction mixture. At last, the cells were stained with 10 mg/ml 3,3′-diaminobenzidine (zsbio, Beijing, China) for 5 min at 37°C, washed with PBS and covered with coverslip. The cells with brown staining on the nucleus were considered to be apoptotic. The percent of apoptotic cells was calculated as following: % (brown cells/the total cells in five fields) and the data were obtained from triplicate experiments.

### CCK8 assay

A total of 1 × 10^4^ cells were seeded in 96-well plate. On the second day, the cells were infected with Ad-LacZ or Ad-PKG II for 24 h. Following serum-free for 12 h, the cells were exposed to l-Arg (2 mM) for 4 h and then treated with EGF (100 ng/ml) for 24 h. Approximately 10 µl of CCK8 dye solution was added to each well and the plate was incubated for 1 h. The optical density (OD) at 450 nm was measured using an ELx800 Absorbance Reader (BioTek Instruments, Inc., Winooski, VT, U.S.A.).

### Migration assay

Transwell plates (Costar, Corning, U.S.A.) were applied to analyze the migration ability, according to the manufacturer’s instructions. Cells were infected with Ad-LacZ and Ad-PKG II for 24 h, respectively. Following serum-free for 12 h, the cells were exposure to l-Arg (2 mM) for 4 h, and then 1 × 10^4^ cells were seeded into the upper chamber containing culture medium without FBS. Cell migration to the bottom side of membrane was induced by medium containing 10% FBS in the lower chamber for 12 h. The cells in the upper chamber were carefully removed with cotton swabs. Migrated cells on the bottom side of the membrane were fixed in 4% paraformaldehyde solution for 30 min, stained in Giemsa solution for 10 min, and then washed. The stained cells were subjected to microscopic examination, and migrated cells were counted in five randomly selected fields per well. All experiments were performed with three replicates.

### Statistical analysis

SPSS13.0 was used for statistical analysis. The data were expressed as mean ± standard deviation (SD). Comparisons between groups were performed using the one-way ANOVA with Tukey’s multiple comparisons test. *P*<0.05 were considered statistically significant.

## Results

### l-Arg-triggered PKG II activation abolished EGF-induced anti-apoptotic effect in OC cells

**Figure 1:**
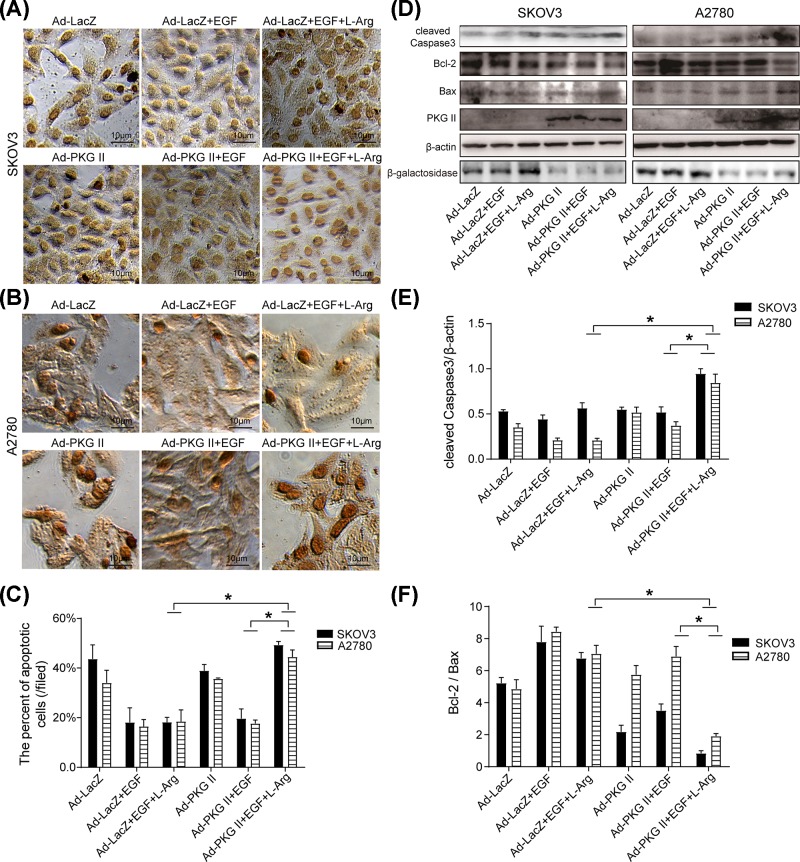
l-Arg-triggered PKG II activation abolished EGF-induced anti-apoptotic effect in OC cells A2780 and SKOV3 cells were infected with Ad-PKG II or Ad-LacZ for 24 h. The infected cells were cultured in serum-free medium for 12 h, stimulated with l-Arg (2 mM) for 4 h, and then treated with EGF (100 ng/ml) for 24 h. (**A,B**) Detection of the apoptosis of the cells by TUNEL method. The cells with brown nuclear staining were considered as apoptotic cells. (**C**) The percent of the apoptotic cells. (**D**) Detection of the expression of PKG II and the apoptosis associated proteins by Western blotting. The cells were harvested and lysed as described in ‘Materials and methods’ section and the cell lysate was subjected to Western blotting for detecting the expression of cleaved Caspase 3, Bcl-2 and Bax. β-galactosidase was detected the LacZ expression. β-actin was as a loading control. (**E,F**) Results of densitometry analysis of the target bands of Western blotting. The results shown were representative images of three independent experiments (*P<0.05).

Apoptosis is a gene-directed program death which is engaged to efficiently eliminate dysfunctional cells, and evasion of apoptosis may induce tumor initiation and therapeutic resistance [27]. TUNEL method was used to analyze the effect of PKG II on apoptosis of OC cells. The results showed that Ad-PKG II combined with l-Arg significantly increased apoptosis compared with corresponding control group (Figure 1A–C); additionally, the cleaved/activated Caspase-3 was also increased (Figure 1D,E); Bcl-2 expression was obviously decreased, conversely, the Bax expression was up-regulated (Figure 1D–F). The above results demonstrated that up-regulated PKG II could be activated by l-Arg; l-Arg-triggered PKG II activation decreased the expression of anti-apoptosis proteins, promoted the expression of pro-apoptosis proteins, inducing SKOV3 and A2780 cells’ apoptosis.

### The proliferation activity of OC cells was blocked by l-Arg-triggered PKG II activation

**Figure 2:**
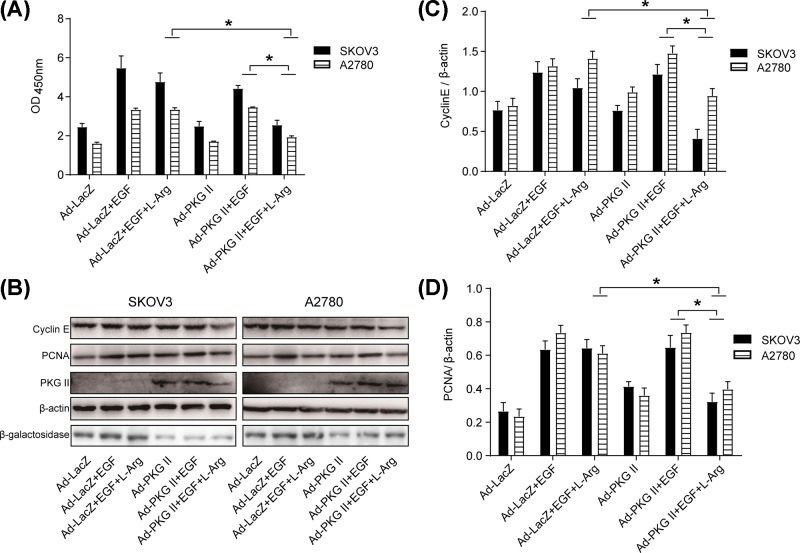
The proliferation of OC cells was inhibited by l-Arg-triggered PKG II activation A2780 and SKOV3 cells were infected as indicated, the infected cells were cultured in serum-free medium for 12 h, stimulated with l-Arg (2 mM) for 4 h, and then treated with EGF (100 ng/ml) for 24 h. (**A**) Detection of the proliferation by CCK-8 assay. The proliferative activities were presented as mean ± SD. (**B**) Detection of the expression of PCNA, Cyclin E, PKG II, β-galactosidase and β-actin by Western blotting. (**C,D**) Results of densitometry analysis of the target bands of Western blotting. The results shown were representative images of three independent experiments (*P<0.05).

Abnormal cell proliferation is associated with multiple pathological status, including cancers. To investigate the effect of l-Arg-triggered PKG II activation on EGF-mediated proliferation of OC cells, cell proliferation activity was analyzed by CCK8 kit, and the expressions of PCNA and Cyclin E were detected by Western blotting. The results showed that l-Arg-triggered PKG II activation efficiently prevented EGF-induced proliferation compared with corresponding control group (Figure 2A). Similarly, l-Arg-triggered PKG II activation significantly inhibited EGF-induced expression of PCNA and Cyclin E in both OC cell lines (Figure 2B–D). These results clearly demonstrated that l-Arg-triggered PKG II activation effectively prevented EGF-induced proliferation of OC cells.

### l-Arg-triggered PKG II activation inhibited the migration activity and regulated the expression of epithelial-to-mesenchymal transition (EMT) associated proteins in OC cells

**Figure 3:**
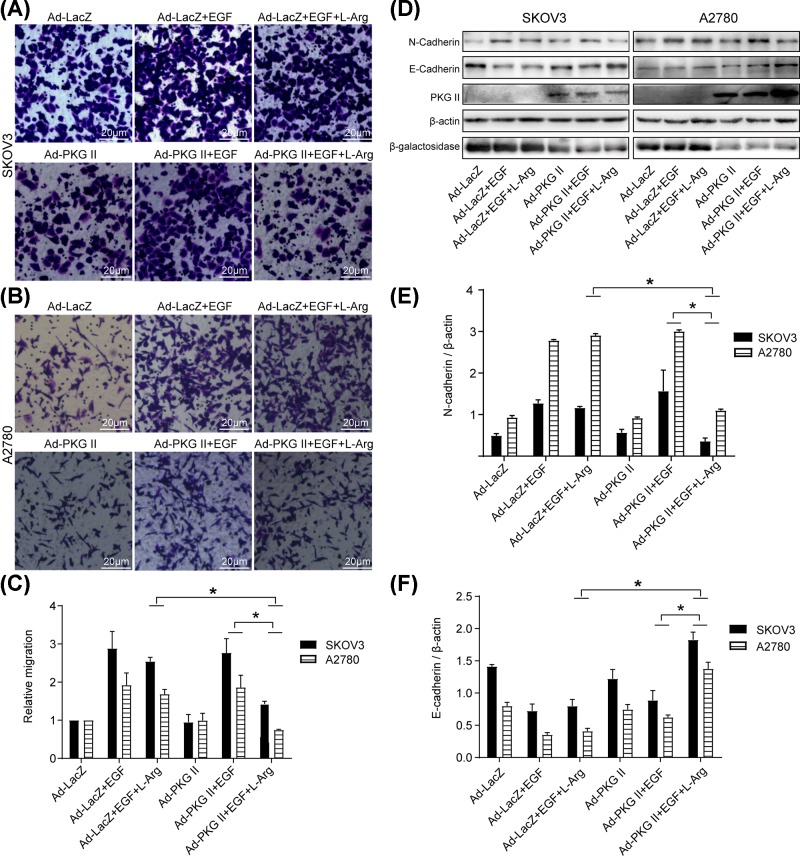
l-Arg-triggered PKG II activation inhibited the migration activity and regulated the expression of EMT-associated proteins A2780 and SKOV3 cells were infected with Ad-PKG II or Ad-LacZ, the infected cells were cultured in serum-free medium for 12 h, stimulated with l-Arg (2 mM) for 4 h. (**A,B**) Analysis of the migration activity of OC cells by Transwell assay. The migration time was 12 h. The representative images of migrated cells stained by Giemsa were shown. (**C**) The relative number of migrated cells in each group (fold vs Ad-LacZ group). The data were shown as the mean ± SD from three independent experiments (*P<0.05). (**D**) Detection of the expression of EMT-associated proteins. After treatment with EGF (100 ng/ml) for 24 h, the cells were harvested and cell lysates were subjected to Western blotting with corresponding antibodies. β-actin was as a loading control. (**E,F**) Results of densitometry analysis of the target bands of Western blotting. The results were representative images of three independent experiments (*P<0.05).

Migration is an important characteristic of cancer cells and contributes to metastasis. The results of transwell migration assay showed that l-Arg-triggered PKG II activation effectively inhibited the migration of OC cells compared with corresponding control group (Figure 3A–C). Additionally, metastasis is a common phenomenon in the progression and dissemination of cancer, and epithelial-to-mesenchymal transition and EMT is also a crucial step [26]. To evaluate the effect of PKG II activated by l-Arg on the expression of EMT-associated proteins, Western blotting was applied and the data showed that the activated PKG II up-regulated the expression of E-Cadherin and decreased the expression of N-Cadherin (Figure 3D–F), indicating that l-Arg-triggered PKG II might inhibit the EMT of OC cells.

### l-Arg-triggered PKG II activation abrogated EGF-induced Raf-MEK and PI3K/Akt signaling pathways

**Figure 4:**
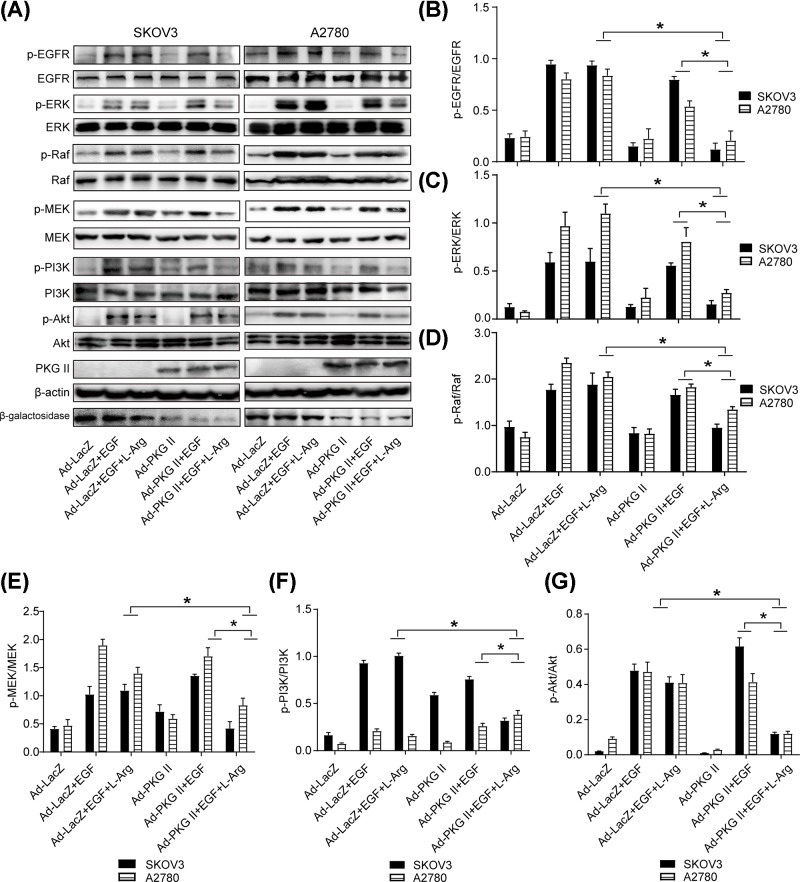
l-Arg-triggered PKG II activation abrogated EGF-triggered Raf-MEK and PI3K/Akt signaling pathways (**A**) Detection of the phosphorylation of EGFR (Tyr^1068^), ERK (Thr^202^/Tyr^204^), c-Raf (Ser^338^), MEK1/2 (Ser^217/221^), PI3K p85 (Tyr^458^) and Akt (Thr^308^). A2780 and SKOV3 cells were infected with Ad-LacZ or Ad-PKG II, the infected cells were cultured in serum-free medium for 12 h, stimulated with l-Arg (2 mM) for 4 h, and then treated with EGF (100 ng/ml) for 5 min. The cells were harvested and the cell lysates were subjected to Western blotting with corresponding antibodies. (**B**–**G**) Results of densitometry analysis of the target bands of Western blotting. The results were representative images of three independent experiments.

Raf-ERK [27] and PI3K/Akt [28] pathways were linked to cancer cells proliferation and transformation, respectively. So, the effect of l-Arg-triggered PKG II activation on the above two pathways were detected. Figure 4 indicated that EGF significantly up-regulated the phosphorylation of EGFR (Tyr^1068^), ERK (Thr^202^/Tyr^204^), c-Raf (Ser^338^), MEK1/2 (Ser^217/221^), PI3K p85 (Tyr^458^), and Akt (Thr^308^). l-Arg-triggered PKG II activation inhibited EGF-induced phosphorylation of the above signaling molecules, suggesting that l-Arg-triggered PKG II activation had inhibitory effect on EGFR activation and EGF-induced Raf-MEK and PI3K/Akt signaling pathways.

## Discussion

OC is the ninth most common malignancy in the world and the fifth most frequent cause of cancer deaths among women [29]. Up to now, OC remains one of the most deadly gynecological malignancies in women [30]. Combination of surgery and chemotherapy is also the conventional therapy, whereas it is poor to control the progression of OC, therefore, it is urgent to find some new therapeutic checkpoints.

PKGs, as serine/threonine kinases, are reported to a play key role in cell migration, invasion, apoptosis and chemoresistance [31]. Our previous results indicated that cGMP-activated PKG II inhibited the growth of gastric cancer cells through blocking EGFR activation [17,32]. Similar results were also observed in some other cancer cells, such as lung cancer, hepatic cancer, renal cancer, colonic cancer, and glioma cell line [33], which showed that PKG II displayed a wide-range of antitumor effects. Furthermore, the recent reports from Fallahian et al.’s [34] lab imply a pro-apoptotic role of PKG in an estrogen receptor-positive (MCF-7) and -negative (MDA-MB-468) breast cancer cell lines. However, it remains unclear whether PKG II could also inhibit OC development. It is well known that PKGs activation could be induced by cGMP, and NO may activate sGC, leading to the increase of intracellular cGMP. NO is released by NO donors or synthesized by NO-synthase (NOS) which converts L-Arg to NO. However, it remains uncertain whether PKG II activation could also be regulated by NO precursor/activator instead of cGMP, such as l-Arg. To clarify the speculation, two OC cells were chosen, and the effects of l-Arg and EGF on the key proteins of cell proliferation, migration, and apoptosis in the above cells were also observed (Supplementary Figure S1). Considering that PKG II should be up-regulated by infecting with adenoviral vector encoding PKG II, Ad-LacZ was applied as a control vector to guarantee that the cells in this group have same background with the cells in experiment group which were infected with PKG II adenoviral vector. In view of this situation, we mainly observed the effect of PKG II with/without l-Arg on cell activities under the premise of infection with the above two vectors. Our results showed that l-Arg could effectively activate overexpressed PKG II, the activated PKG II could inhibit EGF-induced up-expression of PCNA, Cyclin E and Bcl-2, and up-regulated the expression of Bax and cleaved Caspase-3 in OC cells. In addition, OC is more likely to metastasize through intraperitoneal dissemination, furthermore growth factors and proinflammatory cytokines in the tumor microenvironment may increase the metastasis ability through inducing EMT. Collectively, loss of E-cadherin and up-regulation of N-Cadherin are thought to be causative for metastasis [35]. In our study, the results showed that EGF up-regulated the expressions of N-Cadherin, and down-regulated expression of E-Cadherin. However, l-Arg-triggered PKG II activation abolished the above effects, indicating that PKG II could change adhesive properties and make cancer cells to lose mesenchymal abilities. Besides, to invade and metastasize, tumor cells also need to destroy the basement membrane and promote the degradation of extracellular matrix (ECM). Our results suggested that l-Arg-triggered PKG II activation could inhibit the disconnection of intercellular adhesions, prevented the metastasis of OC cells. Preclinical investigations have suggested that the PI3K/Akt and Raf/MEK mediated pathways were frequently activated in OC, and these pathways are considered as attractive candidates for therapeutic interventions [28,36]. Our results also confirmed that l-Arg-triggered PKG II activation blocked Raf/MEK and PI3K/Akt signaling pathways. Furthermore, the activated PKG II could also inhibit the upstream protein EGFR activation which is consistent with previous results [37]. All these data further confirm the inhibitory effect of activated PKG II on OC cells, indicating that PKG II maybe an anti-cancer factor with extensive effects.

## Conclusions

**Figure 5:**
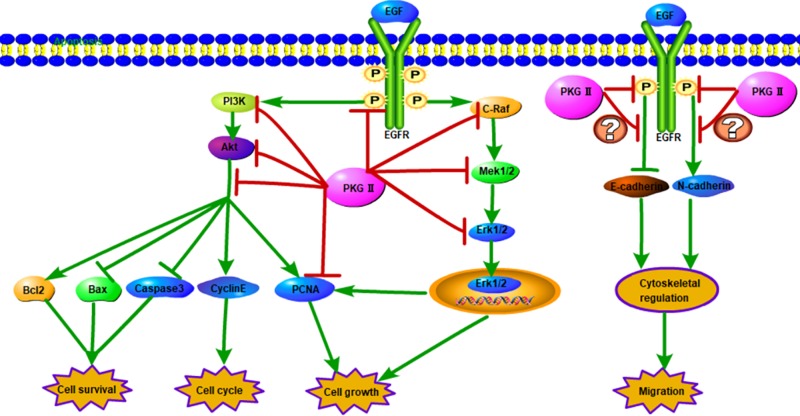
The schematic image of PKG II function in OC cells

In conclusion, l-Arg could effectively activated PKG II and l-Arg-triggered PKG II activation inhibited the proliferation and metastasis, and induced the apoptosis of OC cells via blocking EGF-EGFR-Raf/MEK and PI3K/Akt signal pathways (Figure 5). Therefore, targeted PKG II may be a potential checkpoint for cancer therapy.

## Supporting information

**Supplementary Figure S1:** 

## Abbreviations

Akt: Protein kinase B
Bax: BCL2 associated X
Bcl-2: B-cell lymphoma-2
CCK8: Cell Counting Kit-8
cGMP: cyclic guanosine 3′,5′-monophosphate
DMEM: Dulbecco’s modified Eagle’s medium
ECL: electrochemiluminescence
EGF: epidermal growth factor
EGFR: epidermal growth factor receptor
EMT: epithelial-to-mesenchymal transition
HGF: hepatocyte growth factor
IP: immunoprecipitation
LPA: lysophosphatidic acid
l-Arg: l-arginine
MEK: Mitogen-activated protein kinase kinase
NO: nitric oxide
NOS: NO-synthase
OC: ovarian cancer
PCNA: proliferating cell nuclear antigen
PI3K: phosphatidylinositol 3-kinase
PKG: cGMP-dependent protein kinase
PVDF: polyvinylidene fluoride
Raf: Rapidly Accelerated Fibrosarcoma
sGC: soluble guanylyl cyclase
TUNEL: TdT-mediated dUTP Nick-End Labeling
