# Supplementary figures and images for: Active PKG II inhibited the growth and migration of ovarian cancer cells through blocking Raf/MEK and PI3K/Akt signaling pathways
Source: Biosci Rep. 2019 Aug 13;39(8):BSR20190405. doi: 10.1042/BSR20190405 (PMC6692568; doi:10.1042/BSR20190405)

SKOV3

A2780

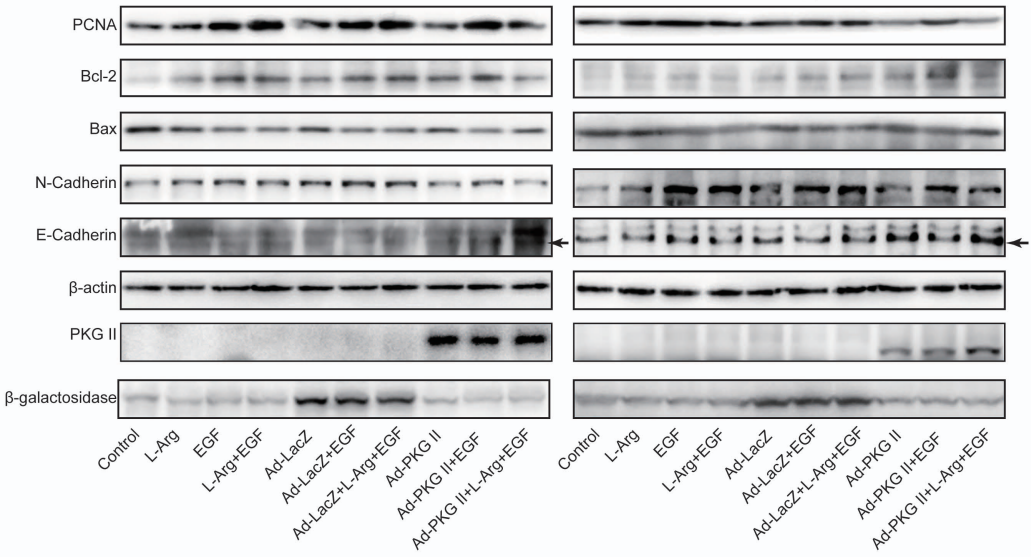

Supplement: Supplementary file 1 [file bsr20190405_Supp1.pdf]
